# Development of the membranous labyrinth in E9.5 to E14.5 C57BL/6N mouse embryos. Stage specific metric and descriptive reference data and their use for identifying malformations

**DOI:** 10.3389/fcell.2026.1725738

**Published:** 2026-04-30

**Authors:** Barbara Maurer-Gesek, Stefan H. Geyer, Wolfgang J. Weninger

**Affiliations:** Division of Anatomy, Center for Anatomy and Cell Biology, Medical University of Vienna, Vienna, Austria

**Keywords:** embryo, imaging, inner ear, mouse model, mutant, phenotyping, topology

## Abstract

Morphological phenotyping of genetically engineered or experimentally challenged mice is the first step in researching the causality of congenital malformations. Especially for spatially complex structures like the inner ear, this is challenging. Our study aims at providing detailed anatomic descriptions and metric information on the membranous labyrinth of mouse embryos from the first demarcation of the otic vesicle to the transition to fetal life. It further intends to use these data as references for objective characterizations of abnormalities in embryos of knock out lines. Using digital data produced from C57BL/6 wild type mouse embryos, 218 surface models of membranous labyrinths were generated and analyzed. The embryo data had been created in the “Deciphering the mechanisms of developmental disorders” program with the aid of “High-resolution episcopic microscopy” and had been staged according to Theiler and Geyer. Detailed anatomic descriptions and measurements of the angles between planes through the semicircular canals and coils of the cochlear duct and the sagittal and horizontal planes as well as measurements of the volumina of the membranous labyrinth and its vestibular, cochlear and endolymphatic components are provided for each developmental stage. This objective metric information was used for diagnosing malformations of the membranous labyrinth of embryos of eight knock-out lines. Our study provides novel information on the normal development of the membranous labyrinth of C57BL/6 embryos, reference data for detailed, objective and stage specific phenotyping of mutants and first results of employing the data for characterizing the spectrum of inner ear malformations in genetically engineered embryos.

## 1 Introduction

Approximately 0,26‰ of newborns suffer from congenital ear malformations (Bartel-Friedrich and Wulke, 2007). Two-thirds are malformations of the outer and middle ear, the components of which originate from the mesenchymal tissues of the first and second pharyngeal arches (Zou, 2023). The remaining one-thirds are defects of the inner ear, which derives from the epidermis of the dorsolateral head. Its first morphologic sign is a small indention, the otic pit. This deepens and forms a vesicle, which sinks into the head mesenchyme and starts growing the semicircular ducts, sacculus, utriculus, cochlear and endolymphatic duct and sac (O'Rahilly et al., 1999).

It is estimated that approximately half of congenital inner ear defects are caused by genetic factors (Wentland et al., 2018). The remaining half are caused by toxins, hypoxia and other extrinsic influences. Investigating their impact on embryo formation requires the use of model organisms. The mouse, as a mammal is the most popular one. Its anatomy and physiology are closely related to humans and several inbred lines with clearly defined genetic backgrounds are available. They permit using loss-of-function (knockout, KO) techniques, forward genetic screens after ENU mutagenesis and other state of the art methods for studying embryo formation, tissue remodeling, and congenital diseases (Brown et al., 2018). Furthermore, they allow to experimentally challenge dams with toxins and other agents and phenotype the offsprings (Kazuki et al., 2016). Whichever approach, the results often are alterations of the morphologic phenotype. Hence, an important step in understanding the influence of genetic, biomechanics and other factors on the genesis of diseases and malformations is the comprehensive and highly detailed assessment of the morphology.

Morphological phenotyping is relatively simple for adults, since sophisticated imaging techniques and detailed atlases are available, which comprehensively describe the normal situation. On the contrary, diagnosing abnormalities of the morphology of embryonic organs is still an enormous challenge due to the small dimensions of embryonic structures and the lack of reference data.

In the late 2000s the High Resolution Episcopic Microscopy (HREM) technique (Weninger et al., 2006) was developed. It produces digital volume data on the basis of episcopic images derived from histological processed and sectioned specimens (Weninger et al., 2006; Geyer et al., 2009; Geyer et al., 2017a). The data comprise of several thousands of block face images and are available a few hours after starting an automated data generation process. The technique proved its value in several studies analyzing genetically engineered mouse embryos (Perez-Garcia et al., 2018; Collins et al., 2019; Adams et al., 2024) and was employed for large scale embryo phenotyping in the “Deciphering the mechanisms of developmental disorders (DMDD)” program. The latter characterized the phenotype of embryos of more than 100 knock out strains producing prenatally lethal individuals (Mohun et al., 2013; Wilson et al., 2016a).

In the scope of DMDD, two fundamental problems of phenotyping organs and structures of embryos became evident. The first is linked to the dynamics of embryogenesis: The developmental progress of single organs varies dramatically within a few hours and mutants are often delayed in their development (Geyer et al., 2017b). This is most obvious at the transition from embryo to fetal life, which in mice is around embryonic day (E) 14. At this stage all major organs remodel dramatically and quickly. Hence, comparing the phenotype of mutants with that of wild types harvested at E14.5 frequently produces false results. To cope with this, staging systems such as that proposed by Theiler (1989) were invented to allow comparison of the phenotypes of embryos of identical developmental maturation. Instead of comparing E14.5 embryos, embryos of Theiler stage (TS) 22 or TS 23 are compared. However, it turned out that embryos of TS22 and embryos of TS23 are still extremely heterogeneous. As a consequence, a novel staging system was implemented for this very period (Geyer et al., 2017b). It distinguishes 5 substages and proved to permit correct diagnosis of malformations and objective diagnosis of delays in embryo maturation.

The second problem that became evident in the scope of DMDD, is the lack of reliable descriptions of the normal morphology and architecture of organs and tissues of embryos and of information covering the full spectrum of norm variations. Thus, DMDD demonstrated the urgent need of reference data, which describe the normal embryo phenotype and its main variations throughout all embryonic stages. Until now, such descriptions do only exist for the cardiovascular system and the cranial nerves in sufficient comprehensiveness (Geyer et al., 2017c; Geyer et al., 2022; Reissig et al., 2022).

To reduce this gap and to aid researching congenital diseases of the auditory and vestibular system, this study aims at creating and presenting highly detailed morphological, topological and volumetric reference data for the developing membranous labyrinth of mouse embryos of TS 15 to TS 21 and the TS 22 and TS 23 substages. It further aims to explore the usefulness of these data by characterizing the phenotype of the membranous labyrinth of selected mouse mutants of the KO-lines *CNOT1, H13, Cfap53, Morc2a, Psph, Rala, Rpgrip1l*, *Smg9*.

## 2 Materials and methods

Using digital volume data produced with the High Resolution Episcopic Microscopy (HREM) method in the scope of the “Deciphering the mechanisms of developmental diseases” (DMDD) program (Mohun et al., 2013), digital surface models of the lumina of the membranous labyrinth and volume models of 109 wild type and eight mutant mouse embryos were created. The DMDD embryos had been bred on the C57BL/6N background and wild type embryos had been harvested at days 9.5–15.5 of embryo development (E). The data of the E9.5 to E13.5 wild type embryos were staged according to Theiler (1989), those of E14.5 and E15.5 were staged in “S” stages according to the system proposed by Geyer et al. (2017b). 10 wild type embryos were of TS15, TS17, TS19, TS21, S22-, S22, S22+, S23- and S23, seven of TS16 and TS20, and five of TS18 were identified in the DMDD data and included in this study. Within the embryos of each developmental stage, no significant differences were observed between the measurements taken from the right and the left sided membranous labyrinths. Building on these results, the groups for all comparative tests between developmental stages were considered with n = 20 for TS15, TS17, TS19, TS21, S22-, S22, S22+, S23- and S23, n = 14 for TS16 and TS20 and n = 10 for TS18. The KO-lines *CNOT1, H13, Cfap53, Morc2a, Psph, Rala, Rpgrip1l*, *Smg9* included one embryo (n = 1) harvested at E14.5 each. They were staged as TS21 (*H13 and Psph*); S22 (*CNOT1, Morc2a, Rpgrip1l and Smg9*) and S22+ (*Cfap53 and Rala*).

**FIGURE 1:**
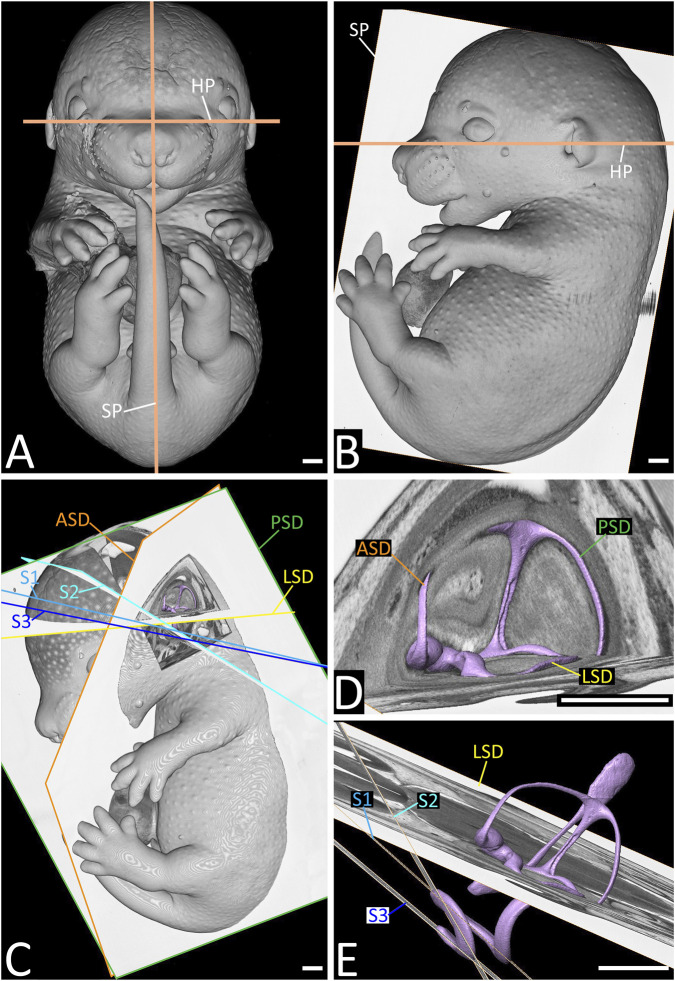
Planes used as a reference for angle measurements. Volume models of embryos **(A–C)** and surface models of the lumen of the inner ear **(C–E)** of a Theiler stage (TS) 23 wild type (WT) embryo from ventral **(A)**, lateral **(B)** and anterolateral **(C–E)**. Note the median-sagittal (SP) and horizontal (HP) planes defined in A and B, and the planes defined by the anterior (ASD, orange), posterior (PSD, green) and lateral (LSD, yellow) semicircular duct, as well as the first (S1, light blue), second (S2, turquoise) and third (S3, dark blue) coil of the cochlear duct, in **(C–E)**. HP is defined by the borders of the lower eyelids and the highest point of the external auditory meatus. Scale bars 500 µm.

Image data processing, segmentation, visualization and analysis were performed in the Amira™ 6.7.0 software package (Thermo-Fisher scientific, Waltham, Massachusetts, United States). A median sagittal plane and a horizontal plane, defined by the borders of the right and left lower eyelid, and the highest point of the opening of the right and the left external auditory meatus were used as reference. The angles between these planes and planes defined by each semicircular duct, the endolymphatic duct, and each coil of the cochlear duct, were measured (Figure 1). The digital surface models were used for measuring the total volume of the lumen of the membranous labyrinth in all wild type and mutant embryos. Additionally, those 3D models were used to calculate the partial volumes of the endolymphatic duct and sac; the cochlear duct and the vestibular apparatus, including utriculus, sacculus, ampullae and semicircular duct in wild type TS19 to S23 embryos and in all mutant embryos.

SPSS statistical software (IBM Corporation, Armonk, NY, United States) and Microsoft Excel (Microsoft Corporation, Albuquerque, NM, United States) were used for descriptive statistics and single factor variance analysis, including Tukey and Games-Howell test were used for comparisons.

## 3 Results

### 3.1 Gross morphology in wild type embryos

**FIGURE 2:**
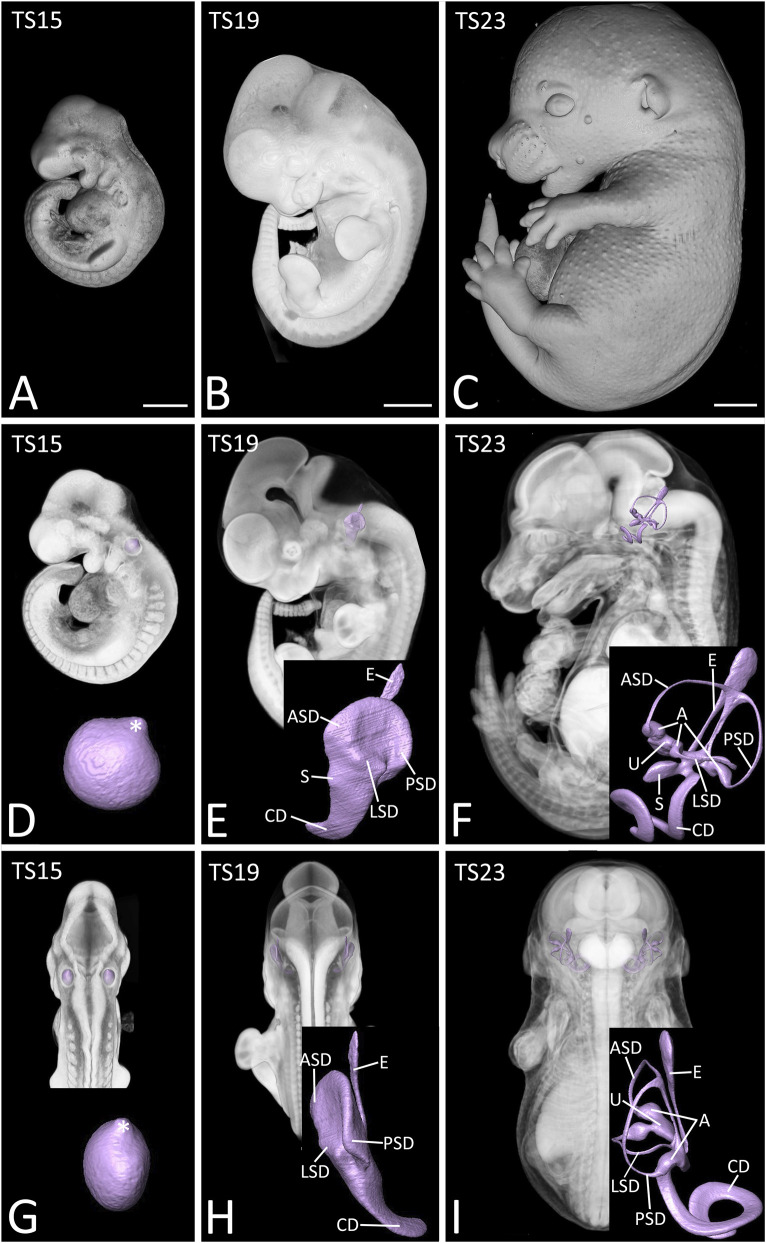
Position of the inner ear in embryos of Theiler stage (TS) 15 **(A,D,G)**, 19 **(B,E,H)** and 23 **(C,F,I)**. Opaque and semitransparent volume models of the embryos and surface models of the lumen of their inner ears from left **(A–F)** and dorsal **(G–I)**. The embryos are arranged along their horizontal plane, as defined by the borders of the lower eyelids and the highest point of the external auditory meatus. Left sided inner ear is displayed in inlays. Forming endolymphatic duct (E, asterisk); forming anterior (ASD), posterior (PSD), and lateral (LSD) semicircular duct; sacculus (S) utriculus (U) ampullae (A) forming cochlear duct (CD). Scale bar 1,000 µm.

**FIGURE 3:**
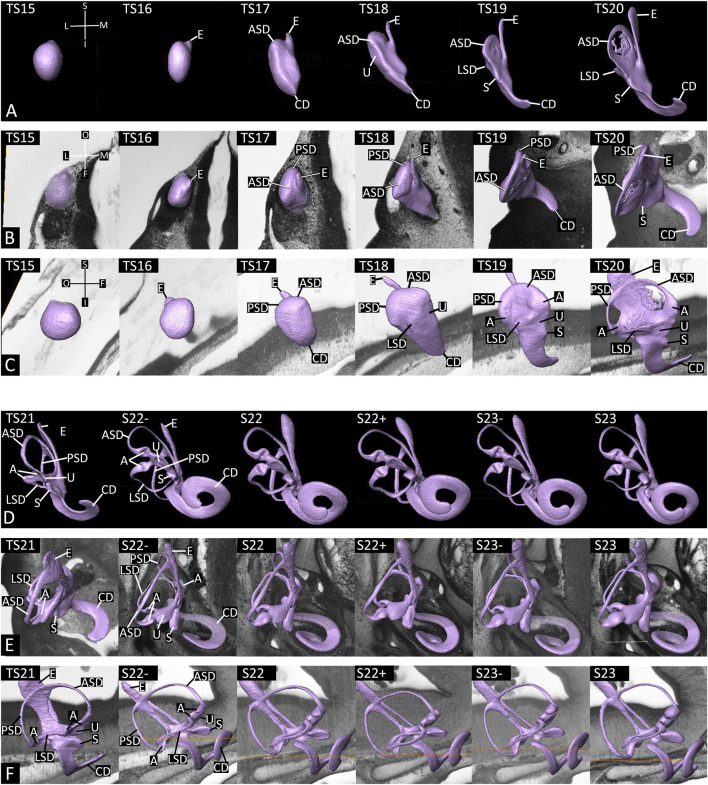
Development of the inner ear from Theiler stage (TS) 15 to TS20 **(A–C)**, and TS21 to TS23; including sub-stages **(D–F)**. Surface models of the lumen of the right inner ear from ventral **(A,D)**, cranial **(B,E)** and lateral **(C,F)**. Note the directional compass; S: superior, I: inferior, L: lateral, M: medial, O: occipital, F: frontal. Alignment in respect to the sagittal and horizontal plane. E: endolymphatic duct, ASD: anterior semicircular duct, PSD: posterior semicircular duct, LSD: lateral semicircular duct, CD: cochlear duct, U: urtriculus, S: sacculus, A: ampullae.

At TS15 a fully closed otic vesicle with a smooth surface was located laterally to the hindbrain inside the head mesoderm. It was an ellipsoid with its main axis approximately in a sagittal plane. At its upper pole a small extension was formed. It extended upwards and represented the first morphological sign of the forming endolymphatic duct (Figures 2, 3).

At TS16 the shape of the vesicle was roughly the same, although the forming endolymphatic duct became slightly more pronounced and smaller with its free tip being directed medially and dorsally (Figure 3).

At TS17 the anterior parts of the, now disc-like inner ear clearly has shifted laterally, a small shift had been also observed in some of the TS16 embryos. The anterior and posterior rims of the vesicle narrowed. The inferior part showed first signs of forming an extension, which eventually will become the cochlear duct. The entire superior part expands causing a relative inferior shift of the endolymphatic duct along the medial surface. This superior expansion is the first sign of the joined anterior and posterior semicircular ducts (Figure 3).

At TS18, the lateral shift of the anterior part of the forming membranous labyrinth and the relative downwards shift of the endolymphatic duct became accentuated. The expansion of the cochlear duct increased. Its tip was directed anteriorly and medially. The narrowing of the anterior and posterior rims continued. Comparisons with later stages revealed that the anterior narrowing is the region at which the utriculus eventually forms. The anterior and posterior semicircular canals become more pronounced and appear as pouches at the lateral-anterior and lateral-posterior superior surface. Comparisons with later stages suggested that these pouches are the region of the forming ampullae. The first sign of the lateral semicircular duct appeared as a small pouch in the center of the lateral wall of the now elongated “vesicle” (Figure 3).

At TS19, the main components of the inner ear, except for the endolymphatic sac were clearly discernible. The endolymphatic duct was still narrow and directed superiorly and posteriorly. The anterior and posterior semicircular canals developed into narrow discs, with the anterior disc being slightly directed laterally in comparison to the posterior. The pouches representing the ampullae were more pronounced and the pouch of the lateral semicircular canal became narrower and oriented obliquely to the horizontal plane. The cochlear duct elongated and formed a crescent with an anteriorly directed apogee. At the anterior connection of the cochlear duct a small pouch, which eventually became the sacculus was discernible (Figures 2, 3).

At TS20 the posterior components of the membranous labyrinth started shifting inferiorly. This process stepwise increased until S23. The inner parts of the originally disc like pouches of the semicircular canals vanished. In three specimens all semicircular canals already appeared as narrow semicircular ducts, except for the sagittal oriented connection of the anterior and posterior one. This was narrow, but broad. In the remaining seven specimens of this group the posterior semicircular canal was visible as a duct, while only parts of the center of the future anterior canal had already vanished and the forming lateral semicircular canal still was a disc like structure. In all specimens of this group, the region of the later ampullae were clearly pronounced pouches. The endolymphatic duct was flat and narrow and had a broad connection to the region inferior to semicircular canals. The sacculus had a pronounced anterior-lateral extension. The cochlear duct coils its tip laterally (Figure 3).

At TS21 all three semicircular ducts were fully developed and more or less in their final relations. The dimension of the connection between the anterior and posterior duct was significantly reduced in its dimension. In the region of the ampullae, the sensory cells were visible as impressions. Inferior to the anterior semicircular duct, the region of the utriculus was discernible. The sacculus was a laterally directed pouch. In six embryos of this group, the cochlear duct started spiraling and formed the first coil. Four embryos had already a distinct second coil. The endolymphatic duct was connected to the region inferior to the semicircular canals and had significantly narrowed. Its tip was broad. This was considered as the first definitive sign of the endolymphatic sac (Figure 3).

At S22-, the semicircular canals had become very thin ducts and the ampullae were clearly visible as thickenings. The sensory cells indented the lumen significantly. The utriculus and sacculus were clearly bordered and the indentions of the sensory cells were visible. The tip of the sacculus was shifted ventrally. The cochlear duct had elongated and formed two coils in all embryos of this group. The tip was directed towards medial, and inferior. The endolymphatic duct had further narrowed and the endolymphatic sac was clearly demarcated (Figure 3).

At S22, the gross morphology of the vestibular part of the membranous labyrinth was relatively similar to S22-. However, the cochlear duct was significantly increased in its length and the tip was directed medially. The endolymphatic duct and sac were clearly demarcated (Figure 3).

At S22+, the morphology of the vestibular part and the endolymphatic duct and sac were similar to S22. However, in four specimens the cochlear duct had already elongated and formed three coils (Figure 3).

At S23-, the morphology of the vestibular part and the endolymphatic duct and sac still was similar to S22. The cochlear duct showed 3 coils in all specimens. Its tip was directed superiorly and medially (Figure 3).

At S23 the morphology of the membranous labyrinth was almost identical to S23- (Figures 2, 3).

### 3.2 Measurements in the wild type embryos

#### 3.2.1 Volume and tortuosity

**FIGURE 4:**
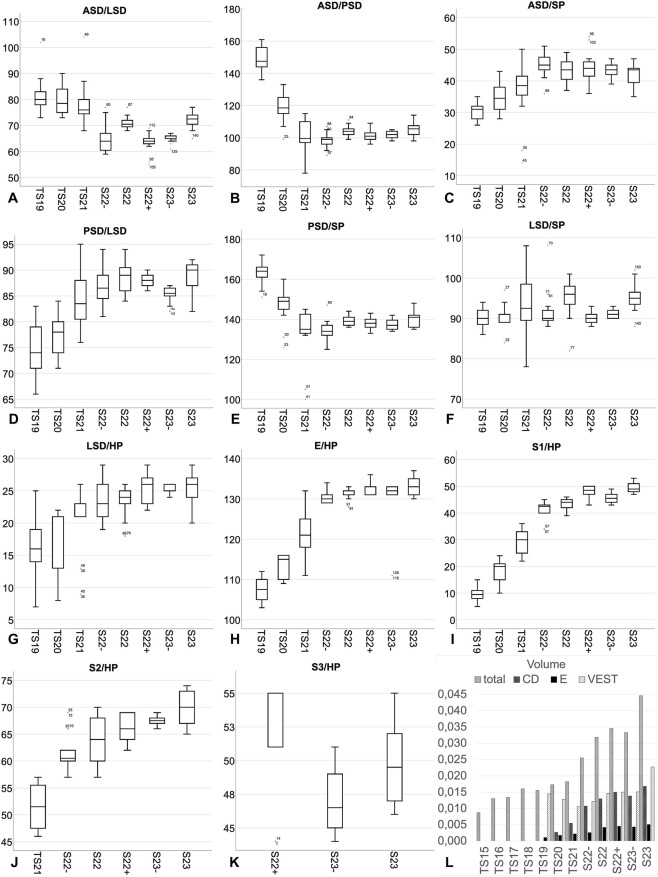
Morphometry of the membranous labyrinth of the inner ear. Angles measured between, **(A)** anterior and lateral semicircular duct (ASD/LSD), **(B)** anterior and posterior semicircular duct (ASD/PSD), **(C)** anterior semicircular duct and sagittal plane (ASD/SP), **(D)** posterior and lateral semicircular duct (PSD/LSD), **(E)** posterior semicircular duct and sagittal plane (PSD/SP), **(F)** lateral semicircular duct and sagittal plane (LSD/SP), **(G)** lateral semicircular duct and horizontal plane (LSD/HP), **(H)** endolymphatic duct and horizontal plane (E/HP), **(I)** first coil of the cochlear duct and horizontal plane (S1/HP), **(J)** second coil of the cochlear duct and horizontal plane (S2/HP), and third coil of the cochlear duct and horizontal plane (S3/HP). **(L)** Total volume (total) of the membranous labyrinth of the inner ear and partial volumes of the endolymphatic duct (E), cochlear duct (CD) and vestibular apparatus (VEST). Volume shown in mm^3^.

The mean volume of the lumen of the otic vesicle respectively membranous labyrinth increased from a mean of 0.010 mm^3^ at TS15 to 0.045 mm^3^ at TS23. Significant leaps in the volumes were observed between TS21 and S22-, and between S23- and S23 (Figure 4).

The volume and tortuosity of the endolymphatic duct and sac could be measured in embryos fromTS19 upwards. The mean volume increased from 0.0011 mm^3^ at TS19 to 0.0051 mm^3^ at S23. Significant leaps in volume were observed between TS20 and TS21, and S22- and S22. The percentage share, which was evident from TS20 upwards, of the mean volume of the endolymphatic duct increased from 10% at TS20% to 12% at S23. The tortuosity of the endolymphatic duct was consistent with 1.1 in all stages.

The volume and tortuosity of the cochlear duct could be measured in embryos from TS20 onwards. The mean volume increased from 0.0026 mm^3^ at TS20 to 0.00618 mm^3^ at S23. Significant leaps were observed between TS20 and TS21, and between TS21 and S22-. The percentage share of the mean volume of the cochlear duct increased from 16% at TS20% to 37% at S23 (Figure 4). The mean tortuosity of the cochlear duct was 1.4 at TS20. It significantly increased between TS21 and S22- then continued increasing continuously to a maximum of 3.29 at S23.

The volume of the vestibular apparatus could be measured from TS19 onwards. It was 0.0145 mm^3^ at TS19, and 0.0227 mm^3^ at S23. The percentage share of the mean volume of the vestibular apparatus decreased from 74% at TS20% to 51% at S23 (Figure 4). The tortuosity of the anterior semicircular duct was between 2.1 and 2.3. The tortuosity of the posterior semicircular duct increased from 2.3 at TS21 to 2.6 at S23 and the tortuosity of the lateral semicircular duct was 2.9 at TS21 and increased to 3.6 in embryos >TS21.

#### 3.2.2 Angles

Angles between the semicircular ducts, between them and the horizontal and sagittal plane, as well as the angles between the endolymphatic duct and the horizontal plane, and the first coil of the cochlear duct and the horizontal plane could be measured in embryos from TS19 onwards. The angle between the second coil and the horizontal plane could be measured from TS21 onwards and that between the third coil and the horizontal plane from S22+ onwards.

The mean angle between the anterior and the lateral semicircular duct varied between 81° at TS19° and 72° at S23. That between the posterior and lateral semicircular duct between 75° at TS19° and 89° at S23 and that between the anterior and posterior semicircular duct between 149° at TS19° and 105° at S2 (Figure 4).

The mean angle between the lateral semicircular duct and the sagittal plane varied between 90° at TS19° and 95° at S23. That between the anterior semicircular duct and the sagittal plane between 31° at TS19, and 42° at TS23 and that between the posterior semicircular duct and the sagittal plane between 163° at TS19, and 140° at S23 (Figure 4).

The mean angle between the lateral semicircular duct and the horizontal plane was 17° at TS19° and 25° at S23.

The mean angle between the endolymphatic duct and the horizontal plane increased from 108° at TS19° to 133° at S23 (Figure 4).

The mean angle between the first coil of the cochlear duct and the horizontal plane increased from 10° at TS19° to 50° at S23. That between the second coil of the cochlear duct and the horizontal plane from 52° at TS21° to 70° at S23 and that between the third coil of the cochlear duct and the horizontal plane was almost identical with 51° and 50° respectively at S22+ and S23 (Figure 4).

### 3.3 Mutant mouse embryos

All mutant mouse embryos had been harvested at E14.5. Staging according to Geyer et al. (2017b) revealed that the *H13* and *Psph* knockouts were of TS21 and thus seriously delayed in their development. The *CNOT1, Smg9, Morc2a* and the *Rpgrip1l* knockouts were of S22, and the *Cfap53* and *Rala* knockouts of S22+. For identifying morphological and metric abnormalities the mutants were compared to the respective group of stage matching wild type embryos.

#### 3.3.1 *H13* TS21

**FIGURE 5:**
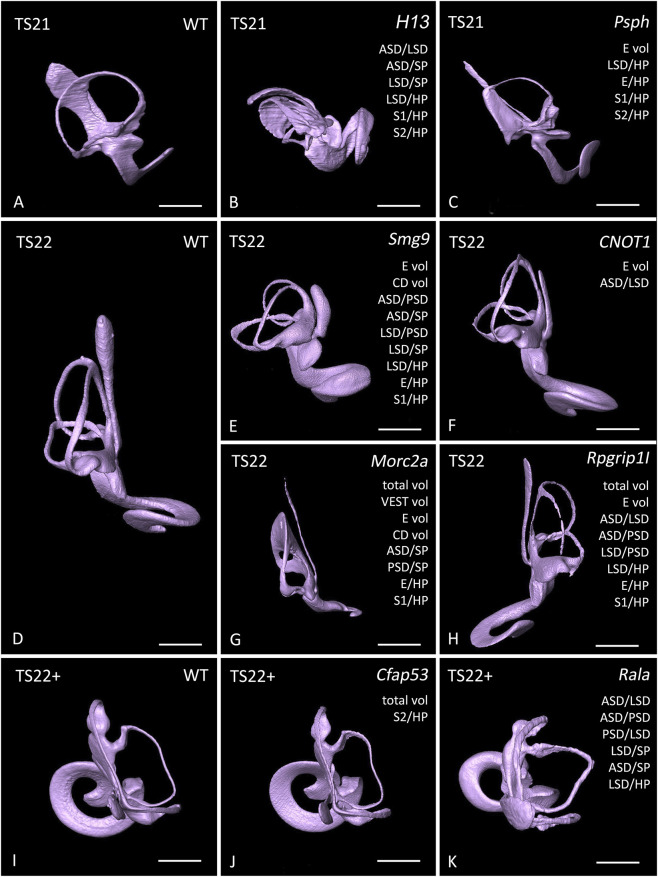
Malformations of the lumen of the inner ear in selected E14.5 embryos of genetically engineered mouse lines producing prenatally lethal offsprings. Comparison of the surface models of stage matching wild types (WT) with that of the mutants. Abnormal features are listed in the respective panels. Note, that 14.5 H13 −/− and Psph −/− mutants are generally delayed in their development and thus matched with Theiler stage (TS) 21 wild type embryos. Total volume of the lumen of the membranous labyrinth of the inner ear (total vol), partial volume of the lumen of the endolymphatic duct (E vol), the cochlear duct (CD vol), and the vestibular apparatus (VEST vol). Angle between anterior and lateral semicircular duct (ASD/LSD), between anterior and posterior semicircular duct (ASD/PSD), between anterior semicircular duct and sagittal plane (ASD/SP); between posterior and lateral semicircular duct (PSD/LSD), between posterior semicircular duct and sagittal plane (PSD/SP); between lateral semicircular duct and sagittal plane (LSD/SP), and horizontal plane (LSD/HP); between endolymphatic duct and horizontal plane (E/HP), and between first (S1/HP) and second (S2/HP) coil of the cochlear duct and horizontal plane. Scale bar 300 µm.

The membranous labyrinth of the right inner ear was visually malformed (Figure 5). Metric data revealed that the total volume of the membranous labyrinth (0.017 mm^3^) and the partial volumes, of the endolymphatic duct (13%), the cochlear duct (38%) and the vestibular apparatus (52%) were in the range of wild type embryos of TS21. Nevertheless, abnormalities were found, in the angle between the anterior and lateral semicircular duct (67°), between the anterior semicircular duct and the sagittal plane (89°), between the lateral semicircular duct and the sagittal plane (141°) and the horizontal plane (60°), between the first coil of the cochlear duct and the horizontal plane (62°) and the second coil of the cochlear duct and the horizontal plane (84°) (Table 1; Supplementary Table S3).

**TABLE 1:** Abnormal angles of the membranous labyrinth in mutants of several knock out lines produced in the DMDD program providing information whether measured values were statistically significant larger ↑ or lower ↓ than in wild types of the same developmental stage.

​	H13_b	Psph	CNOT1	Smg9	Morc2a	Rpgrip1l	Rala	Cfap53
ANGLE°	​	​	​	​	​	​	​	​
ASD/LSD	↓	​	↓	​	​	↓	↑	​
ASD/PSD	​	​	​	↓	​	↓	↓	​
ASD/SP	↑	​	​	↑	↑	​	↑	​
PSD/LSD	​	​	​	↑	​	↓	↑	​
PSD/SP	​	​	​	​	↑	↓	​	​
LSD/SP	↑	​	​	↑	​	​	↑	​
LSD/HP	↑	↑	​	↑	​	↑	↑	​
S1/HP	↑	↑	​	↑	↓	↓	↑	​
S2/HP	↑	↑	​	​	​	​	​	↑
E/HP	​	↑	​	↑	↓	↓	​	​

(ASD) anterior semicircular duct; (LSD) lateral semicircular duct; (PSD) posterior semicircular duct; (SP) sagittal plane; (HP) horizontal plane; (S1) first coil of the cochlear duct; (S2) second coil of the cochlear duct; (E) endolymphatic duct.

#### 3.3.2 *Psph* TS21

The membranous labyrinth of the right inner ear was visually malformed (Figure 5). Metric data revealed that the total volume of the membranous labyrinth (0,012 mm^3^) was at the minimum limit, but within the range of the TS21 wild type embryos, whereas the percentage share of the endolymphatic duct was smaller (3%). All angles, measured in correlation to the horizontal plane, as the lateral semicircular duct (32°), the endolymphatic duct (133°), the first (46°) and the second (66°) coil were larger than the stage matching angles in the wild type embryos. All remaining measurements were within the range of the TS21 wild types (Table 1; Supplementary Table S3).

#### 3.3.3 *Smg9* S22

The membranous labyrinth of the right inner ear was visually malformed (Figure 5). Metric data revealed that the total volume of the inner ear and the partial volume of the vestibular apparatus was within the range calculated in S22 wild types, whereas the partial volume of the cochlear duct was larger than the maximum, and that of the endolymphatic duct was smaller than the minimum calculated in S22 wild types. The measured angles between the posterior and lateral semicircular duct, anterior semicircular duct and sagittal plane, lateral semicircular duct and sagittal plane, lateral semicircular duct and horizontal plane, first coil of the cochlear duct and horizontal plane, and endolymphatic duct and horizontal plane were larger than the maximum, measured in TS22 wild type embryos. The angle measured between anterior and posterior semicircular duct was smaller than the minimum, measured in S22 wild types (Table 1; Supplementary Table S3).

#### 3.3.4 CNOT1 S22

The membranous labyrinth of the right inner ear was visually malformed (Figure 5). Metric data revealed that the total volume of the inner ear, the partial volume of the cochlear duct and that of the vestibular apparatus were within the range of the S22 wild types, whereas the partial volume of the endolymphatic duct (2%) was smaller than the minimum in S22 wild type embryos. Except the measured angle between anterior and lateral semicircular duct (66°), which was smaller than the minimum in S22 wild types, all measured angles were within the range of the S22 wild type embryos (Table 1; Supplementary Table S3).

#### 3.3.5 *Morc2a* S22

The membranous labyrinth of the right inner ear was visually malformed (Figure 5). Metric data revealed that the total volume of the membranous labyrinth (0.02 mm^3^), and the partial volume of the cochlear duct (29°) and the endolymphatic duct (2°) were smaller than the minimum in S22 wild type embryos, whereas the partial volume of the vestibular apparatus (78°) was larger than the maximum in S22 wild type embryos. The lateral semicircular duct was not developed in this ko mouse embryo. Corresponding measurements were not performed. The cochlear duct appeared shortened, more or less straight with a small coil at the tip, pointing laterally. The angle measured between anterior semicircular duct and sagittal plane (60°), and posterior semicircular duct and sagittal plane (158°), were larger than the maximum, the angle between first coil of the cochlear duct and the horizontal plane (27°) and between the endolymphatic duct and the horizontal plane (113°) were smaller than the minimum in S22 wild type embryos. All remaining measurements were within the range of the stage matching wild types (Table 1; Supplementary Table S3).

#### 3.3.6 *Rpgrip1l* S22

The membranous labyrinth of the right inner ear was visually malformed (Figure 5). Metric data revealed that the total volume of the inner ear (0.02 mm^3^) and the partial volume of the endolymphatic duct (8%) were smaller than the minimum in S22 wild types, whereas the partial volume of the cochlea duct and that of the vestibular apparatus was within the range of S22 wild type embryos. The anterior semicircular duct was interrupted at its highest point, and in the area of the ampullae. The measured angle between the anterior and lateral semicircular duct (62°), posterior and lateral semicircular duct (82°), anterior and posterior semicircular duct (97°), posterior semicircular duct and sagittal plane (135°), and first coil of the cochlear duct and horizontal plane (36°) were smaller than the minimum in S22 wild type embryos, whereas the angle between lateral semicircular duct and horizontal plane (28°) was larger than the maximum in S22 wild types. All remaining angular measurements were within the range of S22 wild type embryos (Table 1; Supplementary Table S3).

#### 3.3.7 *Rala* S22+

The membranous labyrinth of the right inner ear was visually malformed (Figure 5). Metric data revealed that the angle between anterior and lateral semicircular duct (75°), between posterior and lateral semicircular duct (99°), between anterior semicircular duct and sagittal plane (79°), between lateral semicircular duct and sagittal plane (127°), between lateral semicircular duct and horizontal plane (48°) and the first coil of the cochlear duct and horizontal plane (52°), were larger than the maximum in S22+ wild type embryos. The measured angle between the anterior and posterior semicircular duct (78°) was smaller than the minimum in S22+ wild types. All remaining measurement results were in the range of S22+ wild type embryos (Table 1; Supplementary Table S3).

#### 3.3.8 Cfap53 S22+

The membranous labyrinth of the right inner ear was visually not malformed (Figure 5). Metric data revealed that the total volume (0,02 mm^3^) of the inner ear was smaller than the minimum in S22+ wild type embryos. The angle measured between the second coil of the cochlear duct and the horizontal plane was larger than the maximum in stage matching wild types. All remaining measurement results were in the range of S22+ wild type embryos (Table 1; Supplementary Table S3).

## 4 Discussion

Our study provides morphologic descriptions and metric information on the membranous labyrinth of mouse embryos from its first appearance until the fetal stage. They are based on a total of 218 data sets from embryos produced on the C57BL/6N background in the scope of the DMDD program (Mohun et al., 2013). DMDD made use of the HREM technique (Weninger et al., 2006). This technique provides 3D information on the basis of episcopic images derived from histological processed and sectioned specimens. The cubic resolution of 3 × 3 × 3 µm^3^ and the almost histological quality allowed us to provide 3D surface models in unmatched detail, which permitted measuring volumes and tortuosity. This significantly expands information deduced from two-dimensional (2D) section images, as presented in groundbreaking classical atlases (Theiler, 1989; Kaufman, 1992), 3D models generated from 2D slices (Chi et al., 2010) and cleared and paint injected specimens (Morsli et al., 1998). Although the latter is capable of providing highly detailed representations of the lumina of the inner ear of all developmental stages (Kiernan, 2006; Bever et al., 2003; Choo et al., 2006), it does not produce real digital 3D data. Measuring angles and distances on such images is error prone and measuring volumes and morphologic complexity is almost impossible.

We used modern staging systems for defining the maturation of the embryos (Theiler, 1989; Geyer et al., 2017b). This adds another dimension to our reference data, since mutants, delayed in their development or mutants which mature slower or faster can be compared to a group of exactly matching wild types. We demonstrate the usefulness of stage specific reference data by phenotyping knock-out embryos of various lines, including such that were delayed in their development. It permitted objective metric definitions of abnormalities in addition to subjective morphologic descriptions.

The combination of highly detailed 3D data and exact staging, permitted highly precise definitions of the steps of normal development of the membranous labyrinth. It enabled us to provide precise information on the timepoints, at which the specific components first become visible and at which they change their appearance significantly. As examples, the endolymphatic duct is the first morphologic structure expanding from the otic vesicle and becomes visible is at TS15, while its sac like termination is not discernible until TS21. The first signs of the cochlear duct appear at TS17 as a small extension at the inferior pole of the otic vesicle. At the same time, the location at which the anterior and posterior semicircular ducts will form is discernible, while the location at which the lateral semicircular duct forms is not discernible until TS18. Beginning with TS20 the semicircular ducts gain their duct-like appearance. The posterior semicircular duct starts, next is the anterior and last the lateral.

A small extension later forming the sacculus and the location or the utriculus are visible from as early as TS19 onwards and at TS21 the sensory cells can be indirectly seen by indentation in these, now prominent structures and the ampullae of the lumen reconstructions.

At developmental stage 22- the inner ear has reached its definitive appearance and the developmental stages 22- to 22+ are largely characterized by further outgrowth of the cochlear duct and continuing increase of the total volume. Interestingly the volume then decreases. This may be explained by the development of the sensory regions at that timepoint (Alsina and Whitfield, 2017), which reduces the luminal volume.

Our data demonstrate that between TS15 and S23 the posterior part of the membranous labyrinth is continuously shifted inferiorly. Additionally, the relative positions of the semicircular ducts to each other change between TS19 to TS21 and the posterior and anterior semicircular ducts become shifted lateral. Additionally, the anterior and the lateral semicircular duct are tilted inferiorly; the anterior stronger, the lateral lesser.

The development of the vestibular apparatus is orchestrated by a large number of genes. Gene knock outs reveal a prominent role of homeobox genes but also of genes, such as *FGF3*, which is essential for the induction of the otic placode; *Dlx5* and *Nkx5.1*, which are important for the morphogenesis of the vestibular apparatus; or *Dlx5*, which is involved in lengthening of the endolymphatic duct and induction of the formation of the semicircular ducts (Hadrys et al., 1998; Matilainen et al., 2007; Merlo et al., 2002). In addition, genes involved in remodeling of the surrounding tissues play essential roles in inner ear formation. Sculpting of the semicircular ducts, for example, depends on *Ntn1* (*Netrin1)* and *fgf9* in the surrounding mesenchyme (Pirvola et al., 2004; Salminen et al., 2000).

We had access to embryos bred in the scope of DMDD. DMDD was a project that aimed at producing comprehensive phenotype-data of prenatally lethal mouse ko-lines. Eight of the DMDD lines were labelled with MP terms associated with abnormal inner ear morphology. We therefore decided to demonstrate the potentials of our reference data for objective diagnosis of inner ear malformations, by reassessing their inner ear phenotype. However, all the assessed mice are no common models for hearing loss or balance disorders. They showed a broad spectrum of severe and life-threatening or lethal malformations with highly variable penetrance (Wilson et al., 2016b). Thus, the presented characterisations do not provide new insights into the mechanisms of inner ear formation. They merely prove the correctness of the subjective diagnosis of inner ear malformations in these lines and demonstrate the value of our reference data for objectively describing these conditions.

Several studies show, that the inner ear morphology, especially that of the semicircular ducts, differs not only between different species of rodents (Hou et al., 2025), but also between different mouse strains and between laboratory and wild mice (Renaud et al., 2024). Consequently, the here presented data have to be used cautiously. However, our results are based on high numbers of mouse embryos from the C57BL/6 inbred strain. This is the most commonly used one, wherefore we are confident, that our data do not only add to understand normal embryogenesis, but also will be of high value for modern biomedical research.

## Data Availability

The datasets presented in this study can be found in online repositories. The names of the repository/repositories and accession number(s) can be found in the article/Supplementary Material.
