# Supplementary material for: Development of the membranous labyrinth in E9.5 to E14.5 C57BL/6N mouse embryos. Stage specific metric and descriptive reference data and their use for identifying malformations
Source: Front Cell Dev Biol. 2026 Apr 30;14:1725738. doi: 10.3389/fcell.2026.1725738 (PMC13171492; doi:10.3389/fcell.2026.1725738)

Supplemental table 1. Mean, minimum and maximum of angle measurements in wild type embryos at the developmental stages 19 to 23. Angles measured between, anterior and lateral semicircular duct (ASD/LSD), anterior and posterior semicircular duct (ASD/PSD), anterior semicircular duct and sagittal plane (ASD/SP), posterior and lateral semicircular duct (PSD/LSD), posterior semicircular duct and sagittal plane (PSD/SP), lateral semicircular duct and sagittal plane (LSD/SP), lateral semicircular duct and horizontal plane (LSD/HP), endolymphatic duct and horizontal plane (E/HP), first coil of the cochlear duct and horizontal plane (S1/HP), second coil of the cochlear duct and horizontal plane (S2/HP; TS21 to S23), and third coil of the cochlear duct and horizontal plane (S3/HP; TS22+ to 23).


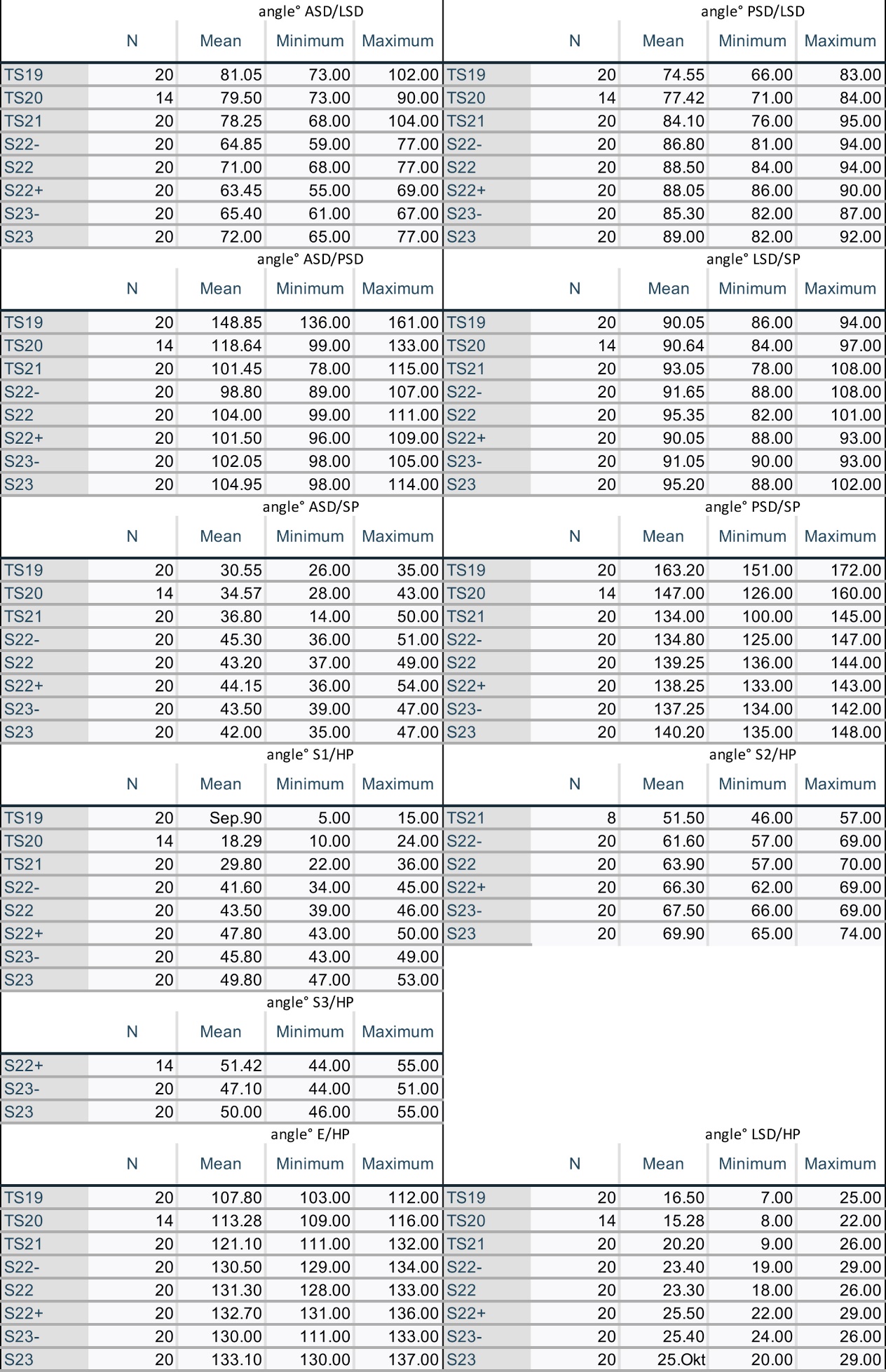


Supplemental table 2. Mean, minimum and maximum of the total volume (total) of the membranous labyrinth of the inner ear calculated in TS15 to S23 wild type embryos, and of the partial volumes of the (E) endolymphatic duct (TS19 to S23), (CD) cochlear duct (TS20 to S23) and (VEST) vestibular apparatus (TS19 to S23). Volume shown in mm^3^. Mean, minimum and maximum of the tortuosity of E, CD and the anterior (ASD), posterior (PSD) and lateral (LSD) semicircular duct.


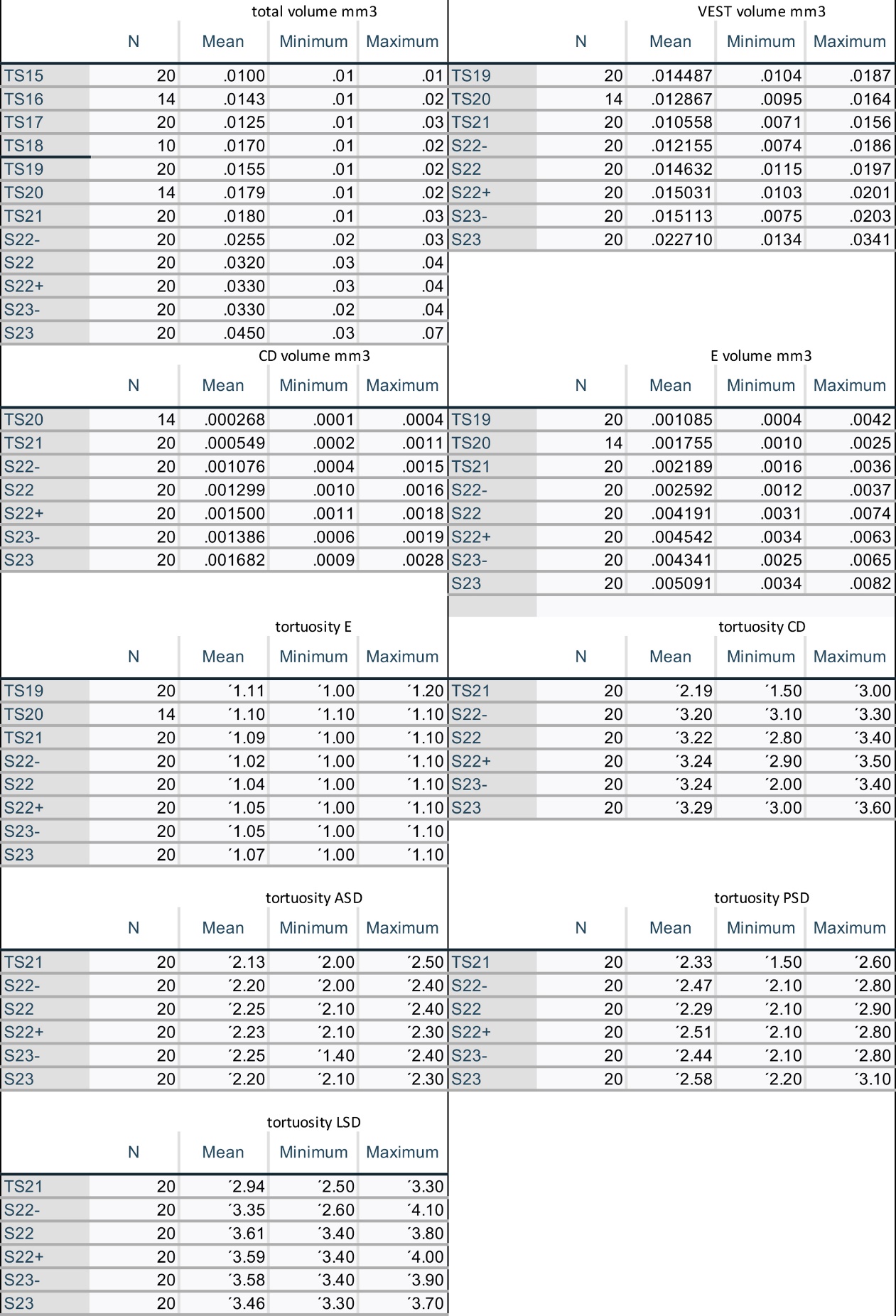


Supplemental Table 3. Angle between , anterior and lateral semicircular duct (ASD/LSD), anterior and posterior semicircular duct (ASD/PSD), anterior semicircular duct and sagittal plane (ASD/SP), posterior and lateral semicircular duct (PSD/LSD), posterior semicircular duct and sagittal plane (PSD/SP), lateral semicircular duct and sagittal plane (LSD/SP), lateral semicircular duct and horizontal plane (LSD/HP), endolymphatic duct and horizontal plane (E/HP), first coil of the cochlear duct and horizontal plane (S1/HP), second coil of the cochlear duct and horizontal plane (S2/HP; TS21 to S23) and calculated total volume (Vol) of the membranous labyrinth of the inner ear, the partial volumes of the (Vol E) endolymphatic duct, (Vol CD) cochlear duct, and ( Vol VEST) vestibular apparatus; measured in mutant embryos *H13* and *Psph* (TS21)*, CNOT1, Smg9, Morc2a* and *Rpgrip1l* (S22)*, Rala and Cfap53* (S22+). Note the range for the listed measurements in stage matching wild type embryos (Range WT). Abnormalities highlighted in blue.


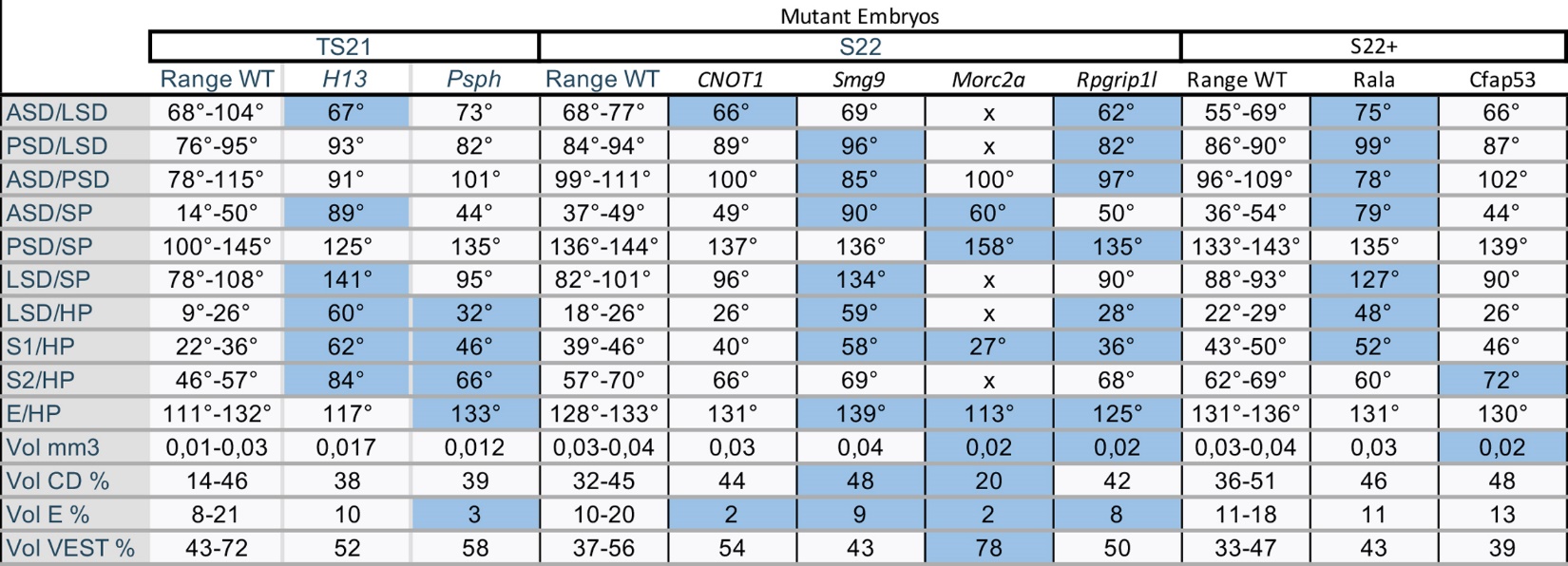

Supplement: Supplementary file 1 [file Table1.docx]
